# A network-guided penalized regression with application to proteomics data

**DOI:** 10.1093/bioadv/vbag038

**Published:** 2026-02-03

**Authors:** Seungjun Ahn, Eun Jeong Oh

**Affiliations:** Department of Population Health Science and Policy, Icahn School of Medicine at Mount Sinai, New York, NY 10029, United States; Tisch Cancer Institute, Icahn School of Medicine at Mount Sinai, New York, NY 10029, United States; Northwell, New Hyde Park, NY 11042, United States; Institute of Health System Science, Feinstein Institutes for Medical Research, Manhasset, NY 11030, United States

## Abstract

**Motivation:**

Network theory has proven invaluable in unraveling complex protein interactions. Previous studies have employed statistical methods rooted in network theory, including the Gaussian graphical model, to infer networks among proteins, identifying hub proteins based on key structural properties of networks such as degree centrality. However, there has been limited research examining a prognostic role of hub proteins on outcomes, while adjusting for clinical covariates in the context of high-dimensional data.

**Results:**

To address this gap, we propose a network-guided penalized regression method. First, we construct a network using the Gaussian graphical model to identify hub proteins. Next, we preserve these identified hub proteins along with clinically relevant factors, while applying adaptive Lasso to non-hub proteins for variable selection. Our network-guided estimators are shown to have variable selection consistency and asymptotic normality. Simulation results suggest that our method produces better results compared to existing methods and demonstrates promise for advancing biomarker identification in proteomics research. Lastly, we apply our method to the Clinical Proteomic Tumor Analysis Consortium (CPTAC) data and identified hub proteins that may serve as prognostic biomarkers for various diseases, including rare genetic disorders and immune checkpoint for cancer immunotherapy.

**Availability and implementation:**

R package is freely available on CRAN repository (https://CRAN.R-project.org/package=NetGreg) and published under General Public License version 3.

## 1 Introduction

Proteins are key components of human cells and are involved in a diverse range of biological functions such as cell division and metabolism. Proteomics is the study of proteins on a large scale (i.e. proteome) and their interactions in a cell (Pandey and Mann 2000). Recent advances in mass spectrometry (MS) technology has enabled simultaneous quantification of multiple protein expressions and identification of protein modification sites for proteomics research (Han *et al.* 2008, Rozanova *et al.* 2021). The MS-based proteomics has been increasingly analyzed for biomarker discovery and disease monitoring in complex human diseases such as cancer (Yang *et al.* 2012, Wisniewski *et al.* 2015, Petralia *et al.* 2024), multiple sclerosis (Åkesson *et al.* 2023), Alzheimer’s disease (Johnson *et al.* 2022), and alcohol-related liver diseases (Niu *et al.* 2022). More importantly, a proliferating number of proteomics studies has spurred development of statistical and bioinformatics methods to analyze the proteomics data. The majority of proteins do not act as independent entities. Instead, they work in concert (i.e. protein interactions) to induce and stabilize a range of cellular and physiological responses that include DNA replication, RNA transcription, protein translation, post-translational modification, targeted degradation, signal transduction, and cell cycle control (Manfredi *et al.* 2019).

The applications of network theory have proven instrumental in inferring the complex landscape of protein interactions through either correlation-based approaches or probabilistic graphical models, similar to other types of -omics disciplines (Shutta *et al.* 2022). Protein interactions can be represented as large interaction networks, wherein nodes symbolize proteins and edges denote pairwise interactions (co-expression), highlighting the presence of hub nodes based on network properties (e.g. proteins with higher degree centrality) (Vella *et al.* 2017). Hub proteins play a pivotal role in maintaining the overall structure of a network. Thus, the removal of hub proteins may lead to a severe deterioration of network connectivity than that of non-hub proteins which has been referred to as centrality-lethality rule (Jeong *et al.* 2001, Barabási and Oltvai 2004, He and Zhang 2006). Furthermore, hub proteins are more likely to be encoded by genes associated with diseases than non-hub proteins (Crua Asensio *et al.* 2017).

Several authors have proposed various methods to estimate protein interactions, either through graphical model estimation or the Weighted Correlation Network Analysis (WGCNA) (Langfelder and Horvath 2008). Among them, Friedman *et al.* (2008) introduced the graphical Lasso to estimate sparse undirected networks, which was subsequently validated using a small proteomics dataset. In related work, Lung Cancer Cohort Consortium (LC3) (2023) performed a graphical Lasso-based network analysis on 36 protein biomarkers for imminent lung cancer diagnosis, demonstrating that the structural arrangement of a network changes according to the disease state (case or matched control) and identifying U-PAR as the central hub protein. On the other hand, Johnson *et al.* (2020) used the WGCNA to identify hub proteins, CD44 and PRDX1, within clusters of densely connected proteins (i.e. modules) which may serve as therapeutic targets for Alzheimer’s disease. Recently, Short *et al.* (2023) fitted linear regressions to correlate continuous brain MRI outcomes and hub proteins identified from the WGCNA (“eigenproteins” as described in the original paper) while accounting for additional clinical covariates such as age, sex, total/HDL cholesterol ratio, and prevalent cardiovascular diseases.

However, the earlier studies (Friedman *et al.* 2008, Johnson *et al.* 2020, Lung Cancer Cohort Consortium (LC3) 2023) have at least two of the following limitations: (i) identified hubs were not adjusted for clinical and demographic covariates when assessing their association with patient outcomes, (ii) the findings are primarily descriptive, and the investigation into the association between hubs and patient health outcomes remains unexplored, thereby limiting the interpretative framework of the study, and (iii) variable selection was not considered, resulting in a suboptimal prediction model characterized by an increased rate of false positives and reduced statistical power. In contrast, Short *et al.* (2023) partially addresses the first two issues by relating hub proteins to MRI outcomes through regression models, but it still lacks a principled variable selection step. This raises a key pertinent question: how should we address the retention of specific proteins variables and clinical covariates that may possess significant clinical and biological relevance, irrespective of their prior identification as hallmark biomarkers or genetic factors in existing studies? This brings into question the rationale for our “network-guided penalized regression.”

Another major challenge in proteomics studies is the high-dimensionality of the covariate space. Recent developments in high-dimensional variable selection approaches include penalized regression methods, such as least absolute shrinkage and selection operator (Lasso) (Tibshirani 1996), adaptive Lasso (Zou 2006), smoothly clipped absolute deviation method (Fan and Li 2001), elastic net (Zou and Hastie 2005), nonnegative garrote (Yuan and Lin 2007), and many others. Recent studies (Villanueva *et al.* 2024, Xu *et al.* 2024) have regressed all proteins in the same prediction model and derived a model with a reduced number of proteins using penalization techniques, including Lasso and elastic net. However, this approach does not consider that a set of proteins interacts with each other as a network. Furthermore, penalizing all proteins is not appropriate when certain variables, such as hubs proteins (for preserving the overall network structure) and clinical covariates (for their clinical importance and potential confounding), should remain in the model. An alternative line of research has attempted to incorporate network structure into the variable selection procedure, as demonstrated by Li and Li (2008) and Huang *et al.* (2011). The method proposed by Li and Li (2008) requires a predefined undirected graph representing known biological relationships, such as pathway structures, which may not be available in all settings, including our application. Meanwhile, Huang *et al.* (2011) introduced the sparse Laplacian shrinkage (SLS) penalty, which combines MCP with a Laplacian quadratic term derived from the graph structure. The SLS method involves non-convex optimization that does not guarantee global optimality and may oversmooth coefficient estimates in densely connected regions, potentially biasing feature selection. Another study by Tutz and Ulbricht (2009) proposed utilizing the correlation between predictors explicitly in the penalty term. However, their method relies on marginal correlations, which may fail to capture the conditional dependencies among variables that underlie network structures. In contrast, our approach uses partial correlations to reflect direct associations while adjusting for the effects of other protein variables, providing a more biologically meaningful representation of molecular networks. More recently, network information has also been incorporated directly into regression models through network-regularized or Bayesian network-guided approaches (Li *et al.* 2019, Ren *et al.* 2024). These methods share the common goal of leveraging network information to improve variable selection and prediction in high dimensional settings, but they do not place an explicit emphasis on distinguishing between hubs and non-hubs, which is often important in proteomic studies.

In the present work, we propose to incorporate network knowledge into variable selection with adaptive Lasso. Specifically, our proposed network-guided penalization procedure retains hub proteins and clinical covariates, while applying an adaptive Lasso penalty to non-hub proteins. The overarching objective of this study is to introduce a method that differentiates hubs from non-hubs and is designed to assess the covariate-adjusted effect of hubs on patient health outcomes with the removal of irrelevant non-hubs. This dual strategy will help preserve the overall network structure by retaining hubs and also enhance the model predictive accuracy by properly adjusting for clinical confounders and penalizing non-hubs for variable selection.

This article is organized into five main sections. Section 1 provides background and motivations. Section 2 covers network estimation, network-guided penalization, and the asymptotic behaviors of the proposed estimators. In Section 3, we present performance metrics from simulation experiments, comparing our method with existing alternatives. In Section 4, we apply our proposed method to proteomics data from the National Cancer Institute (NCI) Clinical Proteomic Tumor Analysis Consortium (CPTAC). Finally, we wrap up our discussion by addressing challenges, limitations, and future directions in Section 5.

## 2 Methods

Consider a finite population of *n* subjects. Let *Y* be an outcome of interest, $X=\{X_{1},\ldots ,X_{p}\}$ be a vector of proteins, $Z=\{Z_{1},\ldots ,Z_{c}\}$ be a vector of potential confounders that need to be adjusted in the regression, such as age, gender, and other related diseases and conditions at baseline. In our data example, X is high-dimensional, whereas the dimension of Z is low or moderate.

### 2.1 Network estimation with sparse Gaussian graphical model

Our idea is built upon the Gaussian graphical model (GGM) (Lauritzen 1996) to estimate a PPI network, where an edge represents conditional dependency of a pair of nodes (proteins) after controlling for all other nodes in a network. In a GGM network, the weight of an edge is the partial correlation and represent whether or not and how strongly the two nodes co-occur. Thus, the network structure is decided by an estimation of partial correlations.

We assume X follows a multivariate normal distribution:


$$X∼N_{p}(\mu ,\Sigma ),$$

where $\mu =(\mu_{1},\ldots ,\mu_{p})$ is a vector of means for each protein and $\Sigma ={\left[\Sigma_{jk}\right]}_{1\leq j,k\leq p}$ is a p×p variance-covariance matrix that is positive definite. The inverse of Σ is a precision matrix (or concentration matrix), denoted by $\Sigma^{-1}=\Theta ={\left[\theta_{jk}\right]}_{1\leq j,k\leq p}$. The off-diagonal elements of precision matrix can be standardized with a sign reversal to calculate the partial correlation of two proteins $X_{j}$ and $X_{k}$, conditional on all other proteins in X (Shutta *et al.* 2022), which is our focus in this section,


(1) $$\rho_{X_{j},X_{k}|X_{-j,-k}}=\frac{-\theta_{jk}}{\sqrt{\theta_{jj}\theta_{kk}}},$$

where $X_{-j,-k}$ is a set of proteins without *j* and *k*, and $\theta_{jk}$ denotes the corresponding element of Θ. Furthermore, Θ can be constructed as a network with protein nodes that are connected by edges when $\rho_{X_{j},X_{k}|X_{-j,-k}}\neq 0$.

As in most “-omics” disciplines, there are generally hundreds of samples, while each sample has thousands of proteins (Wang *et al.* 2021). In the high-dimensional setting where n≪p, a maximum likelihood estimation of Σ may not be accurate due to singularity (i.e. det(Σ)=0) (Kuismin and Sillanpää 2017). Thereby, a range of regularization methods have been proposed to estimate a sparse GGM, which bypass the issue of n≪p and non-invertible Σ (Meinshausen and Bühlmann 2006, Friedman *et al.* 2008, Peng *et al.* 2009).

In the present paper, we consider the graphical Lasso (GL) (Friedman *et al.* 2008) to impose a sparsity on GGM by penalizing $\ell_{1}$-norm of the elements of Θ. The GL estimates Θ by maximizing the following penalized log-likelihood:


(2) $$\hat{\Theta }=\underset{\Theta }{\text{argmax}}\{\;\text{log}\;\det(\Theta )-\mathrm{tr}(\hat{\Sigma }\Theta )-\lambda ||\Theta |{|}_{1}\},$$

where $\hat{\Sigma }$ is an empirical variance-covariance matrix, $\left||\Theta |{|}_{1}=\sum_{j\neq k}|\theta_{j,k}\right|$ denotes the sum of absolute value of edges, and λ denotes a tuning parameter. The optimal λ can be chosen based on the extended Bayesian information criterion (eBIC) (Foygel and Drton 2010). The eBIC is expressed as


**Figure 1:**
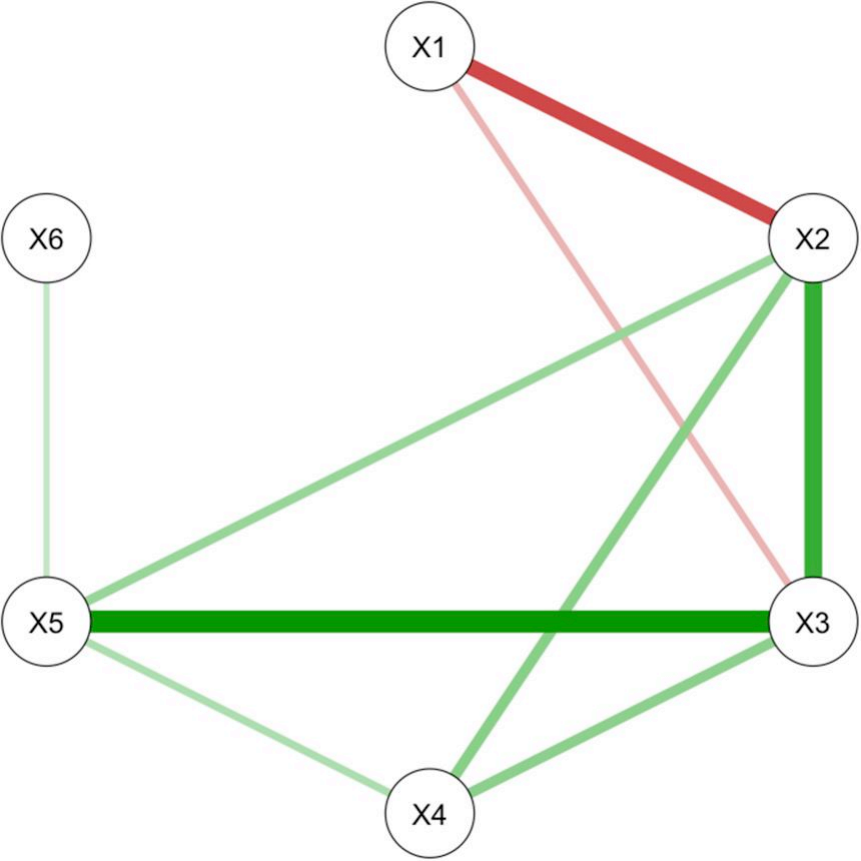
An example network plot to visualize a network with p=6 proteins based on partial correlation estimates from a graphical Lasso algorithm in combination with an extended Bayesian information criterion. Any missing edge between nodes (e.g. $X_{1}-X_{4}$, $X_{1}-X_{5}$, and $X_{1}-X_{6}$) corresponds to a partial correlation estimates of exactly zero in $\hat{\Theta }$.

eBIC=−2ℓ(Θ)+E log(n)+4γE log(p),

where ℓ(Θ) is a penalized log-likelihood to estimate $\hat{\Theta }$ in (2), *E* is a number of edges or non-zero elements of Θ, and γ∈[0,1] denotes a non-negative eBIC hyperparameter. Of note, the eBIC becomes an ordinary BIC when γ=0 and a higher value of γ leads to a greater sparseness by removing more edges (Shutta *et al.* 2022). Following (1), ${\hat{\rho }}_{jk}$’s are calculated based on the elements of resulted $\hat{\Theta }$. See Fig. 1 for an example of the network constructed as described.

The network centrality has been studied to measure the extent of biological or topological importance that a node has in a network (Junker and Schreiber 2008, Ashtiani *et al.* 2018). For each protein *k*, the network centrality (degree centrality in continuous scale; ${\hat{\phi }}_{k}$) is calculated as the marginal sum of the association matrix.


$${\hat{\phi }}_{k}=\sum_{j=1}^{p}|{\hat{\rho }}_{jk}|,$$

where k=1,…,p. We define protein nodes with higher ${\hat{\phi }}_{k}$ relative to others as hub proteins, where the number of hub proteins h<p depends on user-specified parameters, δ and τ, by taking h=min(⌊pδ⌋,τ). More details are discussed in the following subsection.

### 2.2 Network-guided $\ell_{1}$-penalization

Suppose we observe data from *n* individuals. For each individual, the data is of the form {Z,X,Y}. When the goal is to regress Z,X on *Y*, the following model is usually considered:


(3) Y=μ+Zζ+Xη+ϵ,

where μ is the intercept, ζ and η are the coefficients for Z and X, respectively, and ϵ is the error component assumed to be normally distributed around zero with constant variance $\sigma^{2}$.

In this project, we decompose X into two parts: hub proteins, denoted by $H\in R^{h}$, and non-hub proteins, denoted by $N=X\setminus H\in R^{q}$, such that $\eta =(\eta_{1},\eta_{2})$, where $\eta_{1}$ and $\eta_{2}$ are the coefficients of H and N, respectively. The network-guided $\ell_{1}$-penalization procedure aims to adjust the level of penalization on non-hub proteins $N\in R^{q}$, while preserving hub proteins and potential confounders along with unpenalized intercept, denoted by $U=(1,Z,H)\in R^{t}$, where t=h+c+1, in the model to be adjusted for. Thus, model (3) can be re-written as


(4) $$\begin{array}{c} Y=\mu +Z\zeta +(H,N){\left(\eta_{1},\eta_{2}\right)}^{T}+\epsilon \\ \;=(1,Z,H){\left(\mu ,\zeta ,\eta_{1}\right)}^{T}+N\eta_{2}+\epsilon \\ \;=U\alpha +N\beta +\epsilon , \end{array}$$

where $\alpha =(\mu ,\zeta ,\eta_{1})$ and $\beta =\eta_{2}$ are the corresponding coefficients for U and N, respectively.

To deal with high-dimensional data, we propose a regression approach with a Lasso-type penalty. The network-guided $\ell_{1}$-penalization estimates $\left({\hat{\alpha }}_{n},{\hat{\beta }}_{n}\right)$ are obtained by minimizing the following objective function:


$$L_{n}(\alpha ,\beta )=∥Y-U\alpha -N\beta {∥}_{2}^{2}+\lambda_{n}\sum_{j=1}^{q}w_{j}|\beta_{j}|,$$

where $\lambda_{n}$ is a non-negative tuning parameter that controls model complexity and $w_{j}\geq 0$ is the weight for adjusting the level of penalization on $\beta_{j}$. In this project, we apply adaptive Lasso (Zou 2006) to shrink coefficients of non-hub proteins such that only significant ones remain in the model, while keeping hub proteins and clinical covariates. In adaptive Lasso, the weight vector is defined as $\hat{w}=|{\tilde{\beta }}_{n}{|}^{-\nu }$ for some ν>0, where ${\tilde{\beta }}_{n}$ is any root-*n*-consistent estimator. This imposes heavier penalties on covariates with smaller coefficients. In practice, we use perturbed elastic net estimates for ${\tilde{\beta }}_{n}$, following Zou and Zhang (2009). The 5- or 10-fold cross-validation can be used to select an optimal pair of $\left(\nu ,\lambda_{n}\right)$.

**Remarks.** The formula h=min(⌊pδ⌋,τ) which is used to identify H helps control that the number of hub proteins depends on a user-specified proportion to the size of X with the pre-specified positive constant τ. In this study, we set τ=⌊(p+20)/16⌋, such that the dimension of non-penalized terms is moderate. It is essential that starting with the minimal size of H is desired due to the nature of a partial penalization. Even if all essential proteins were not classified as H, they would still undergo evaluation as N through a penalization method and could remain in the final model if they are shown to be predictive of outcomes in a data-driven manner. Namely, with a lower δ, we may miss some important hubs, but they will still go through the penalization step, which gives them an opportunity to be recovered. On the other hand, with a larger δ, it is possible to include hubs with zero signals, which can be difficult to exclude later. For this reason, a lower δ is safer to explore.

We assume the following two regularity conditions:

(A1) $\epsilon \overset{\Delta }{=}Y-U\alpha_{0}-N\beta_{0}$ has mean zero and finite variance $\sigma^{2}$, and is independent of (U,N).

(A2) $n^{-1}{\left(U,N\right)}^{T}(U,N)\to C$, where C is positive definite.

Let $J=\left\{j:\beta_{0j}\neq 0,j=1,\ldots ,q\right\}$ be the true active set of variables in N, and assume that |J|=r<q. Denote the estimated active set of variables by ${\hat{J}}_{n}=\left\{j:{\hat{\beta }}_{nj}\neq 0,j=1,\ldots ,q\right\}$. Let $\beta_{0J}=\{\beta_{0j}:j\in J\}$ and ${\hat{\beta }}_{nJ}=\{{\hat{\beta }}_{nj}:j\in J\}$. Denote $\theta ={\left(\alpha^{T},\beta^{T}\right)}^{T}$ for any $\alpha \in R^{t}$, $\beta \in R^{q}$. Then $S=\left\{1,2,\ldots ,t\right\}\cup \left\{s:\theta_{0s}\neq 0,s=t+1,\ldots ,t+q\right\}$ is the true active set of variables in (U,N), and thus J is always the subset of S. Denote $C_{S}\in R^{\left(t+r)\times (t+r\right)}$ is the top-left block matrix (i.e. sub-matrix) of $C\in R^{\left(t+q)\times (t+q\right)}$. In the following, we demonstrate the oracle property of our estimators.

Theorem 1. *Suppose* $\lambda_{n}=o(\sqrt{n})$  *and* $\lambda_{n}n^{(\nu -1)/2}\to \infty$*. Then under model (4) and regularity conditions (A1)–(A2), the network-guided adaptive Lasso estimators satisfy the following properties:*
 

- *(variable selection consistency)* $\underset{n}{\lim}P({\hat{J}}_{n}=J)=1$,
- *(joint asymptotic normality)*
 
  $$\sqrt{n}\left(\begin{array}{c} {\hat{\alpha }}_{n}-\alpha_{0}\\ {\hat{\beta }}_{nJ}-\beta_{0J} \end{array}\right)\to_{d}\;N(0,\sigma^{2}C_{S}^{-1}).$$

The theorem above implies that the network-guided adaptive Lasso estimators enjoys variable selection consistency and asymptotic normality. The proof is deferred to the Supplementary Material.

## 3 Simulation experiments

In this section, simulation studies are conducted to compare the proposed network-guided (NG) adaptive Lasso estimators with other existing alternatives and evaluate model performance using various metrics. We make a comparison with the adaptive Lasso (aLasso), Lasso, elastic net (enet), and ridge regression models. These methods do not distinguish between proteins when applying penalties and therefore serve as natural comparison methods for our work. Additional comparison methods include the correlation-based penalized estimators (CBPE) proposed by Tutz and Ulbricht (2009) and the sparse Laplacian shrinkage (SLS) method introduced by Huang *et al.* (2011). For each method, the 5-fold cross-validation was used to select the optimal tuning parameters.

### 3.1 Settings

We generated X from the multivariate normal distribution $N_{p}(0,\Sigma )$ with the correlation structure $\Sigma ={\left[\Sigma_{jk}\right]}_{1\leq j,k\leq p}$, where $\Sigma_{jk}$ is 1 if j=k, 0.9 if j∈{1,2,3,4}≠k, and $0.9^{\left|j-k\right|}$ if j∈{5,…,p}≠k. Three potential confounders were generated as follows: $Z_{1}∼U(0,1)$, $Z_{2}∼\text{Bernoulli}(0.25)$, and $Z_{3}∼\text{Bernoulli}(0.65)$. The outcome variable was generated according to the model (3) with σ=1, μ=0.5, ζ=(2.5,2.5,2.5) along with the two different scenarios for η:

1. Strong signal: $\eta =(3.5_{5},0_{5},-1.5_{5},0_{p-15})$
2. Weak signal: $\eta =(1,-0.8,0.6,0,0,-1.5,-0.5,1.2,0_{p-8})$

Different combinations of sample size and dimension (network size), denoted as (n,p) = (50,60), (100,60), (100,300), were considered, representing Setting I, II, and III, respectively. Following the terms used in Monti and Filzmoser (2021), each sample size/dimension combination represents moderate-high-dimensional, low-high-dimensional, and high-dimensional setting, respectively. The high-dimensional setting is often observed in proteomics studies, as in most “-omics” disciplines. For each setting, we repeated the simulation 100×.

### 3.2 Performance metrics

The predictive model performance is mainly evaluated using the root-mean-squared error (RMSE) and calibration slope (CSL). Overall variable selection performance was assessed by the F1 score, defined as


$$F1\;\text{score}=\frac{2\cdot \text{TP}}{2\cdot \text{TP}+\text{FP}+\text{FN}},$$

and the Matthews correlation coefficient (MCC) proposed by Matthews (1975), defined as


$$\text{MCC}=\frac{\text{TP}\cdot \text{TN}-\text{FP}\cdot \text{FN}}{\sqrt{\left(\text{TP}+\text{FP})\cdot (\text{TP}+\text{FN})\cdot (\text{TN}+\text{FP}\;)\cdot (\text{TN}+\text{FN}\right)}},$$

where TP, TN, FP, and FN are true positives (the number of nonzero variables correctly selected), true negatives (the number of zero variables correctly excluded), false positives (the number of zero variables incorrectly included), and false negatives (the number of nonzero variables left out of the model), respectively.

### 3.3 Simulation results

Tables 1 and 2 present model performance metrics under various settings based on 100 simulation replicates. Performance measures are evaluated on an independent test set of size 1000. For each metric, the mean value is reported along with the standard deviation in parentheses. The average computational time in seconds is also provided. In the strong signal case (Table 1), with different specifications of δ, our proposed NG method consistently outperformed other existing methods, in terms of lower RMSE, better calibration, higher F1 score, and higher MCC. Especially under Setting II, aLasso and our NG method performed well, in terms of calibration slope nearly close to an ideal value of 1 and a very high MCC.

**Table 1:** Simulation results under strong signal case using network-guided (NG) method, adaptive Lasso (aLasso), Lasso, elastic net (enet), ridge regression, correlation-based penalized estimators (CBPE), and sparse Laplacian shrinkage (SLS) method*.

Setting	*n*	*p*	Method**	RMSE	CSL	F1 score	MCC	Avg. runtime (sec)
I	50	60	NG (δ=0.06)**	1.33 (0.59)	**1.01** (0.01)	0.81 (0.11)	0.75 (0.15)	0.61
			NG (δ=0.08)	**1.32** (0.57)	**1.01** (0.01)	0.81 (0.11)	0.77 (0.14)	0.61
			NG (δ=0.10)	**1.32** (0.63)	**1.01** (0.01)	0.81 (0.10)	0.76 (0.13)	0.61
			aLasso	2.89 (0.78)	1.04 (0.03)	0.71 (0.15)	0.66 (0.19)	0.04
			Lasso	1.88 (0.71)	1.03 (0.02)	0.64 (0.10)	0.56 (0.14)	0.02
			enet	2.22 (0.58)	1.04 (0.02)	0.52 (0.07)	0.40 (0.11)	0.02
			ridge	8.16 (0.72)	1.63 (0.14)	0.34 (0.00)		0.02
			CBPE	2.77 (0.31)	1.04 (0.03)	0.34 (0.00)		0.36
			SLS	7.15 (2.06)	0.75 (0.07)	**0.89** (0.11)	**0.87** (0.14)	0.24
II	100	60	NG (δ=0.06)	**0.67** (0.10)	**1.01** (0.00)	**0.99** (0.03)	**0.98** (0.03)	0.60
			NG (δ=0.08)	0.68 (0.11)	**1.01** (0.00)	0.98 (0.03)	**0.98** (0.03)	0.60
			NG (δ=0.10)	0.70 (0.11)	**1.01** (0.00)	0.95 (0.03)	0.94 (0.04)	0.60
			aLasso	0.70 (0.10)	**1.01** (0.00)	0.98 (0.04)	**0.98** (0.04)	0.02
			Lasso	0.74 (0.12)	1.02 (0.00)	0.71 (0.07)	0.65 (0.08)	0.01
			enet	0.89 (0.14)	1.02 (0.00)	0.51 (0.05)	0.41 (0.07)	0.01
			ridge	0.94 (0.11)	1.02 (0.01)	0.34 (0.00)		0.02
			CBPE	1.57 (0.17)	1.02 (0.01)	0.34 (0.00)		0.19
			SLS	7.15 (1.73)	0.73 (0.05)	0.94 (0.05)	0.93 (0.06)	0.28
III	100	300	NG (δ=0.01)	**1.08** (0.11)	**1.00** (0.00)	**0.97** (0.03)	**0.97** (0.03)	1.05
			NG (δ=0.02)	1.15 (0.13)	**1.00** (0.00)	0.88 (0.03)	0.87 (0.03)	1.05
			NG (δ=0.03)	1.19 (0.13)	**1.00** (0.00)	0.79 (0.03)	0.78 (0.03)	1.06
			aLasso	2.42 (0.46)	1.02 (0.01)	0.85 (0.06)	0.86 (0.05)	0.03
			Lasso	1.15 (0.21)	1.02 (0.00)	0.92 (0.06)	0.92 (0.05)	0.02
			enet	1.22 (0.23)	1.02 (0.00)	0.85 (0.07)	0.85 (0.07)	0.03
			ridge	9.87 (0.90)	1.45 (0.07)	0.08 (0.00)		0.13
			CBPE	5.29 (0.33)	1.07 (0.02)	0.08 (0.00)		8.83
			SLS	26.5 (7.73)	0.54 (0.13)	0.86 (0.28)	0.85 (0.34)	1.32

* The best results are highlighted in boldface.

** δ is the proportion used for the number of hub protein nodes in a network.

**Table 2:** Simulation results under weak signal case using network-guided (NG) method, adaptive Lasso (aLasso), Lasso, elastic net (enet), ridge regression, correlation-based penalized estimators (CBPE), and sparse Laplacian shrinkage (SLS) method*.

Setting	*n*	*p*	Method**	RMSE	CSL	F1 score	MCC	Avg. runtime (sec)
I	50	60	NG ()	0.26 (0.10)	**1.01** (0.02)	**0.88** (0.08)	**0.86** (0.09)	0.59
			NG (δ=0.08)	0.23 (0.10)	**1.01** (0.02)	0.87 (0.07)	0.85 (0.08)	0.59
			NG (δ=0.10)	**0.20** (0.08)	**1.01** (0.01)	0.86 (0.06)	0.83 (0.06)	0.59
			aLasso	0.49 (0.11)	1.07 (0.04)	0.80 (0.05)	0.79 (0.06)	0.03
			Lasso	0.34 (0.14)	1.05 (0.03)	0.62 (0.09)	0.58 (0.11)	0.02
			enet	0.46 (0.14)	1.07 (0.04)	0.53 (0.07)	0.48 (0.09)	0.02
			ridge	2.06 (0.11)	3.43 (3.41)	0.25 (0.00)		0.02
			CBPE	0.89 (0.12)	0.97 (0.04)	0.25 (0.00)		0.34
			SLS	1.89 (0.27)	1.27 (0.16)	0.67 (0.13)	0.62 (0.14)	0.20
II	100	60	NG (δ=0.06)	**0.08** (0.01)	1.01 (0.00)	0.96 (0.02)	0.96 (0.03)	0.59
			NG (δ=0.08)	**0.08** (0.01)	**1.00** (0.00)	0.95 (0.01)	0.94 (0.01)	0.59
			NG (δ=0.10)	**0.08** (0.01)	**1.00** (0.00)	0.91 (0.02)	0.89 (0.02)	0.59
			aLasso	**0.08** (0.01)	1.01 (0.00)	**1.00** (0.00)	**1.00** (0.00)	0.02
			Lasso	**0.08** (0.01)	1.02 (0.00)	0.78 (0.07)	0.77 (0.07)	0.01
			enet	0.09 (0.01)	1.02 (0.00)	0.64 (0.07)	0.62 (0.07)	0.01
			ridge	0.54 (0.05)	1.10 (0.03)	0.25 (0.00)		0.02
			CBPE	0.36 (0.04)	0.96 (0.01)	0.25 (0.00)		0.21
			SLS	1.82 (0.17)	1.23 (0.09)	0.75 (0.08)	0.71 (0.09)	0.26
III	100	300	NG (δ=0.01)	0.28 (0.08)	**1.01** (0.01)	**0.87** (0.07)	**0.87** (0.07)	1.04
			NG (δ=0.02)	**0.19** (0.06)	**1.01** (0.01)	0.81 (0.02)	0.80 (0.03)	1.05
			NG (δ=0.03)	0.20 (0.07)	**1.01** (0.01)	0.70 (0.02)	0.71 (0.03)	1.05
			aLasso	0.45 (0.06)	1.05 (0.02)	0.81 (0.03)	0.82 (0.03)	0.05
			Lasso	0.46 (0.05)	1.06 (0.02)	0.44 (0.09)	0.47 (0.07)	0.03
			enet	0.53 (0.07)	1.08 (0.03)	0.33 (0.06)	0.39 (0.05)	0.03
			ridge	2.14 (0.12)	1.83 (0.60)	0.06 (0.00)		0.14
			CBPE	2.48 (0.22)	0.73 (0.07)	0.06 (0.00)		8.78
			SLS	2.37 (0.25)	0.66 (0.07)	0.67 (0.12)	0.68 (0.10)	1.12

* The best results are highlighted in boldface.

** δ is the proportion used for the number of hub protein nodes in a network.

However, it is worth noting that in Settings I or III where the number of proteins is greater or significantly greater than the number of observations, our NG method showed much better performances than the alternative methods. For example, in Setting I, the NG method had a F1 score of 0.81, which was higher than the rest of the methods, ranging from 0.34 (ridge or CBPE) to 0.71 (aLasso). Furthermore, the proposed NG method demonstrated a smaller standard deviation of RMSE, compared to the competing methods. Under Setting III, the RMSE standard deviations were 0.11 (NG, δ=0.01) and 0.13 (NG, δ=0.02 or δ=0.03), which were smaller than 0.90 (ridge) or 0.46 (aLasso). Additionally, ridge, enet, and CBPE showed poor performance, characterized by one or more of the following: high RMSE, calibration slope far from 1, low F1 score, or low MCC. It is also worth noting that the SLS method showed a high RMSE, with the highest RMSE observed particularly in Setting III, as well as poor calibration across all settings. Although the F1 score and MCC of the SLS method were high in Setting I, the overall combination of performance metrics suggests that the resulting coefficient estimates were likely biased.

Similarly, in the weak signal case (Table 2), our proposed NG method continued to outperform the other methods. For instance, in Setting III, the RMSE of the NG method with δ=0.02 was 0.19, which was less than half of that of aLasso (RMSE, 0.45), Lasso (RMSE, 0.46), and enet (RMSE, 0.53), and almost one-tenth of ridge (RMSE, 2.14), CBPE (RMSE, 2.48), or SLS (RMSE, 2.37). In all settings, the NG method showed lower RMSE, better calibration, higher F1 score, and higher MCC.

In all cases, our method consistently performed well with a small δ. As discussed in Section 2.2, proteins that may have been initially missed out based our network estimation can still be included in the final model through variable selection if they show predictive potential, thereby demonstrating good overall model performance. The computational time for our method tends to depend more on *p* than on *n*, since the hub identification process is affected when *p* is large. Although some conventional methods were computationally efficient, such as Lasso and ridge, in settings with larger *p* (e.g. Setting III), our NG method was relatively faster than the CBPE and SLS methods. It is also worth noticing that the proposed NG method, which incorporates prior network information by excluding hubs from penalization, consistently outperformed aLasso in terms of lower RMSE, better calibration, higher F1 score, and higher MCC, particularly in settings where *p* exceeds *n*, as in Settings I and III. This suggests that selectively relaxing penalization on proteins identified as hubs, which are often functionally important in biological networks, allows the model to better preserve relevant signals while shrinking noise, leading to improved prediction performance when the parameter space is large relative to the sample size.

## 4 Real data application

### 4.1 Clinical proteomic tumor analysis consortium data

A pre-processed MS-based proteomics data of the National Cancer Institute (NCI) Clinical Proteomic Tumor Analysis Consortium (CPTAC) was downloaded from the Proteomic Data Commons (PDC; https://pdc.cancer.gov/pdc/cptac-pancancer), which is one of the largest public repositories of proteogenomic data. In this paper, the Estimation of STromal and Immune cells in MAlignant Tumor tissues using Expression data (ESTIMATE) (Yoshihara *et al.* 2013) score was obtained from the CPTAC metadata, and is the outcome of interest. The ESTIMATE score is a sum of the scores of immune and stromal cells, the two main non-tumor components in the tumor microenvironment. It has been used in a variety of cancer studies such as osteosarcoma (Zhang *et al.* 2020), head-and-neck squamous cell carcinoma (HNSCC) (Liu *et al.* 2023), and lung cancer (Lehtiö *et al.* 2021). The higher the score, the lower the purity of the tumor. The clinical covariates that are included in the modeling with proteins are age, sex, body mass index (BMI), cancer staging, and size of tumor.

### 4.2 Analysis results

We analyzed 337 gene-level proteins that are subsets of B cell-immune module from 108 patients with HNSCC. B cell-immune module was the rarest that accounts only in 3% of identified modules depicted in a recent study (Petralia *et al.* 2024). Of note, B cells have the ability to promote humoral immunity through the production of antibodies and its presence has been associated with responses to immunotherapy in cancer studies (Garaud *et al.* 2019, Ruffin *et al.* 2021).

**Figure 2:**
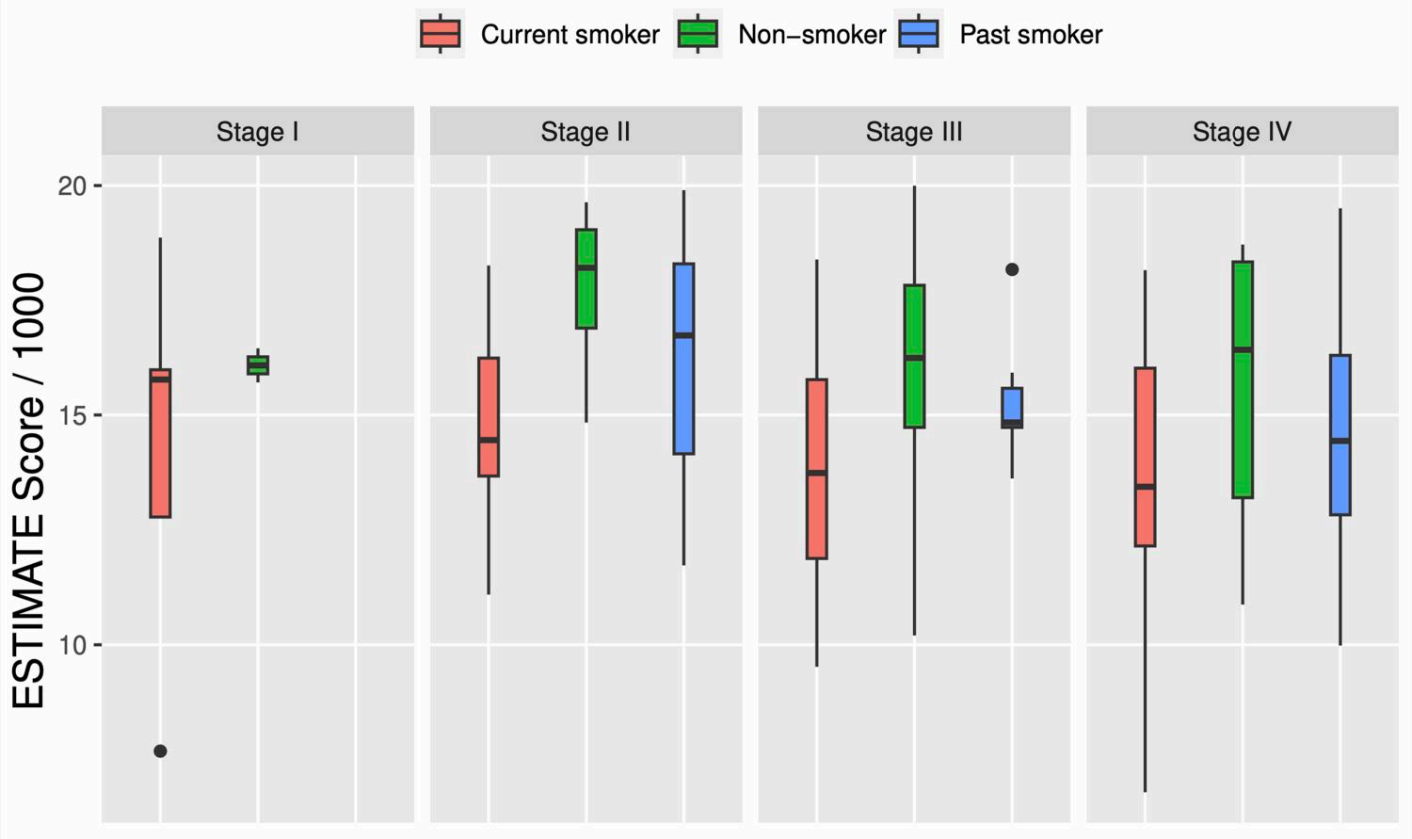
Side-by-side boxplots to visualize the distributions of scaled ESTIMATE scores (scores divided by 1000) of CPTAC-HNSCC patients (n=108) by smoking status and cancer staging.

Table 3 summarizes characteristics of the full cohort sample, and we stratified these characteristics by smoking status. The study samples comprised older adults (median [IQR] = 62.0 [11.3]), predominantly male (87%), and normal weights (24.0 [5.9]), according to the Centers for Disease Control and Prevention (Weir and Jan 2023). Patients (70.4%) were found in tumors staged III or IV with the median size of tumor was 3.2 cm (IQR = 1.8). The median ESTIMATE scores were significantly different across smoking statuses according to a Kruskal–Wallis test. In general, non-smokers had higher ESTIMATE scores in all cancer stages from early (stage I) to advanced disease (stage IV), when compared with current and past smokers. This is shown as side-by-side box plots in Fig. 2.

**Table 3:** Patient characteristics of CPTAC-HNSCC patients.

Characteristics	Overall (*N* = 108)	Current smoker (*N* = 61)	Non-smoker (*N* = 20)	Past smoker (*N* = 27)	*P* value^a^
Age in years^b^	62.0 (11.3)	62.0 (12.0)	59.5 (11.3)	64.0 (9.0)	.2
Sex^c^					.005
Female	14 (13.0%)	8 (13.1%)	6 (30.0%)	0 (0.0%)	
Male	94 (87.0%)	53 (86.9%)	14 (70.0%)	27 (100.0%)	
BMI^b^	24.0 (5.9)	24.0 (5.2)	24.6 (6.2)	24.0 (5.7)	.005
Cancer staging^c^					.7
Stage I	7 (6.5%)	5 (8.2%)	2 (10.0%)	0 (0.0%)	
Stage II	25 (23.1%)	14 (23.0%)	4 (20.0%)	7 (25.9%)	
Stage III	30 (27.8%)	16 (26.2%)	7 (35.0%)	7 (25.9%)	
Stage IV	46 (42.6%)	26 (42.6)	7 (35.0%)	13 (48.1)	
Tumor size in cm^b^	3.2 (1.8)	3.0 (2.0)	3.1 (1.4)	4.0 (1.3)	.5
ESTIMATE score^d^	14.8 (4.0)	13.9 (3.7)	16.4 (3.5)	15.2 (3.4)	.005

a Kruskal–Wallis test or Fisher’s exact test as appropriate.

b Median (IQR).

c n(%) .

d Scaled (score divided by 1000).

We used 75% of randomly selected data samples for model fitting, and the remaining 25% data for model evaluation. Further, our proposed method was benchmarked on the CPTAC-HNSCC data against other popular methods that were evaluated in our simulation experiments above. When applying our method, three different values were considered for δ, the proportion of hub proteins in a network. We hypothesize that 3 (δ=0.01), 6 (δ=0.02), and 10 (δ=0.03) out of 337 gene-level proteins from B-cell immune module are defined as hubs in a network. As a whole, Table 4 shows that our NG method had lower RMSE and better calibration slope (closer to 1) than that of the benchmark models. We also repeated 100 random train/test splits (Supplementary Table 1, available as supplementary data at *Bioinformatics Advances* online), and the results consistently showed that the NG method outperformed the competing methods in terms of lower RMSE and better calibration.

**Table 4:** Performance results of methods applied to CPTAC-HNSCC patients*.

	RMSE	CSL
NG (δ=0.01)	1.95	**0.95**
NG (δ=0.02)	1.78	**0.95**
NG (δ=0.03)	**1.77**	0.89
aLasso	1.80	1.07
Lasso	2.01	1.88
Ridge	2.27	2.27
enet	1.90	1.51
CBPE	2.94	0.56
SLS	2.23	2.27

* The best results are highlighted in boldface.

δ is the proportion used for the number of hub protein nodes in a network.

In addition to benchmark results, the HUGO Gene Nomenclature Committee (HGNC)-approved symbols of hub proteins are listed in Table 5. Their estimated effects, which correspond to the coefficient estimates from the fitted penalized model, are provided in Supplementary Table 5, available as supplementary data at *Bioinformatics Advances* online. HGNC-approved symbols are protein-coding gene annotations for each known human gene (Seal *et al.* 2023). In this analysis, PABPC1, LGALS1, and GIMAP7 were found in common between three different values for δ parameter. By searching through integrative databases of human genes [GeneCards (Stelzer *et al.* 2016)] and human diseases [MalaCards (Rappaport *et al.* 2017)], PABPC1 is linked to viral diseases that are transmitted by mosquitoes such as rift valley fever and dengue virus. LGALS1 is related to corneal ulcer. Interestingly, a recent study (Qin *et al.* 2022) suggested that GIMAP7 has a potential as a prognostic biomarker and immune checkpoint gene for immunotherapy in pan-cancer. Additional hub proteins were identified when increasing the size of δ. BLNK is associated with a rare genetic immunodeficiency disorder, called autosomal agammaglobulinemia (Cardenas-Morales and Hernandez-Trujillo 2022).

**Table 5:** A complete list of hub proteins identified through the proposed method^a^.

	HGNC approved symbols of hub proteins
NG (δ=0.01)	**PABPC1**, **LGALS1**, **GIMAP7**
NG (δ=0.02)	**PABPC1**, **LGALS1**, **GIMAP7**, MEN1, RPLP1, HNRNPD
NG (δ=0.03)	**PABPC1**, **LGALS1**, **GIMAP7**, MEN1, RPLP1, HNRNPD, CASP10, BLNK, SDC1, MUC4

a Results are shown by varying sizes of the proportion of hub proteins, δ. Listed proteins are mapped to gene symbols approved by the HUGO Gene Nomenclature Committee (HGNC). Proteins that are found in common between all three δ values are boldfaced.

## 5 Discussion

In this study, we have proposed a network-guided penalized regression model that retains hub proteins and clinical covariates, applying an adaptive Lasso penalty exclusively to non-hub proteins. This model screens out irrelevant non-hub proteins based on their predictive value, while maintaining key variables, including potential confounders for their clinical importance and hub proteins that are identified through network estimation. Our hybrid method leverages network estimation and variable selection through partial penalization, representing a novel approach. We have also shown that our network-guided estimators enjoy variable selection consistency and asymptotic normality. In contrast to WGCNA’s soft-thresholding approach that promotes a scale-free topology, our method constructs networks based on partial correlations and penalization via graphical Lasso, allowing hubs to emerge from strong conditional dependencies rather than from enforced topological constraints.

Through a series of simulation studies and real data application, we have observed that the proposed network-guided approach demonstrates good overall performance measures. It is noteworthy that our method shines particularly in a finite sample high-dimensional setting, where the number of proteins significantly exceeds the number of observations, which is a common scenario in most “-omics” disciplines. In addition, as shown in the simulations, lower δ generally resulted in better variable selection performance in terms of F1 score and MCC, while higher δ tended to produce lower values in these metrics. This is because increasing the value of δ and expanding the number of hubs can lead to the inclusion of additional proteins as hubs that may have no signal. To reduce the risk of misspecification, it is generally recommended to start with a lower δ. Although some important hubs may be missed initially with a lower δ, they can still pass through the penalization step and potentially be recovered.

Incorporating hub proteins is crucial, as they may serve as prognostic biomarkers across diverse diseases, including rare genetic disorders and immune checkpoints in cancer immunotherapy. To address this, we retain hub proteins in our network guided penalization framework to evaluate their covariate-adjusted associations with patient outcomes. The partial penalization allows the model to explain as much outcome variability as possible through unpenalized terms, with remaining variability captured by non-hubs, which are of lower clinical focus. Retaining hub proteins also helps preserve the underlying network structure. Removing hubs is more likely to disrupt this structure than removing non-hubs, so treating hubs as unpenalized terms helps maintain proteins that are essential for understanding both the outcome of interest and the overall network structure.

Hub nodes can be identified based on various network properties. Here, although we have opted for degree centrality due to its intuitive nature and popularity in the literature, it may also be useful to choose the centrality measure that biologically aligns with the disease of interest or specific conditions. This can be done by selecting the measure that is commonly used for that disease or by working closely with clinical experts. To assess the impact of different centrality measures in the proposed approach, we conducted sensitivity analyses in the simulation studies, using betweenness centrality in Supplenmentary Tables 2 and 3 and eigenvector centrality in Supplementary Tables 4 and 5, available as supplementary data at *Bioinformatics Advances* online. The results were largely consistent, indicating that the proposed NG method consistently outperformed the competing approaches. This consistency is likely attributable to the fact that the hubs identified using different network properties did not differ substantially, as evidenced by some overlap among hubs across different network properties, as shown in Supplementary Fig. 1, available as supplementary data at *Bioinformatics Advances* online. For future studies, it would be valuable to evaluate the parameters in our proposed approach as being determined through data-driven optimization rather than being user specified. This could involve determining which objective function is most appropriate to optimize and assessing how these choices influence the results. However, even under a data-driven framework, clinically meaningful upper bounds may still be necessary, since a very high proportion of features classified as hubs is unlikely to be practical in proteomic studies. It is also of interest to consider more flexible regression approaches which could handle repeatedly measured proteins data and/or complex relationships (e.g. non-linear or piece-wise) between covariates and the outcome of interest.

## Supplementary Material

vbag038_Supplementary_Data

## Data Availability

The pre-processed proteomics and clinical data (metadata) from the National Cancer Institute-initiated CPTAC are available in the Proteomic Data Commons (https://pdc.cancer.gov/pdc/cptac-pancancer). The NetGreg R package is freely available in the Comprehensive R Archive Network (CRAN) repository (https://cran.r-project.org/web/packages/NetGreg/index.html). Please reach out to the corresponding author (Seungjun Ahn, seungjun.ahn@mountsinai.org) if you have any further inquiries.

## References

[vbag038-B1] Ashtiani M , Salehzadeh-Yazdi A, Razaghi-Moghadam Z et al A systematic survey of centrality measures for protein-protein interaction networks. BMC Syst Biol 2018;12:80. 30064421 10.1186/s12918-018-0598-2 PMC6069823

[vbag038-B2] Barabási A , Oltvai Z. Network biology: understanding the cell’s functional organization. Nat Rev Genet 2004;5:101–13. 14735121 10.1038/nrg1272

[vbag038-B3] Cardenas-Morales M , Hernandez-Trujillo V. Agammaglobulinemia: from X-linked to autosomal forms of disease. Clin Rev Allergy Immunol 2022;63:22–35. 34241796 10.1007/s12016-021-08870-5 PMC8269404

[vbag038-B4] Crua Asensio N , Muñoz Giner E, de Groot N et al Centrality in the host-pathogen interactome is associated with pathogen fitness during infection. Nat Commun 2017;8:14092. 28090086 10.1038/ncomms14092 PMC5241799

[vbag038-B5] Fan J , Li R. Variable selection via nonconcave penalized likelihood and its oracle properties. J Am Stat Assoc 2001;96:1348–60.

[vbag038-B6] Foygel R , Drton M. Extended Bayesian information criteria for gaussian graphical models. Adv Neural Inf Process Syst 2010;23:604–12.

[vbag038-B7] Friedman J , Hastie T, Tibshirani R. Sparse inverse covariance estimation with the graphical lasso. Biostatistics 2008;9:432–41. 18079126 10.1093/biostatistics/kxm045 PMC3019769

[vbag038-B8] Garaud S , Buisseret L, Solinas C et al Tumor infiltrating b-cells signal functional humoral immune responses in breast cancer. JCI Insight 2019;5:e129641. 31408436 10.1172/jci.insight.129641 PMC6795287

[vbag038-B9] Han X , Aslanian A, Yates J III. Mass spectrometry for proteomics. Curr Opin Chem Biol 2008;12:483–90. 18718552 10.1016/j.cbpa.2008.07.024 PMC2642903

[vbag038-B10] He X , Zhang J. Why do hubs tend to be essential in protein networks? PLoS Genet 2006;2:e88. 16751849 10.1371/journal.pgen.0020088 PMC1473040

[vbag038-B11] Huang J , Ma S, Li H et al The sparse Laplacian shrinkage estimator for high-dimensional regression. Ann Stat 2011;39:2021–46. 22102764 10.1214/11-aos897 PMC3217586

[vbag038-B12] Jeong H , Mason S, Barabási A et al Lethality and centrality in protein networks. Nature 2001;411:41–2. 11333967 10.1038/35075138

[vbag038-B13] Johnson ECB , Carter EK, Dammer EB et al Large-scale deep multi-layer analysis of Alzheimer’s disease brain reveals strong proteomic disease-related changes not observed at the RNA level. Nat Neurosci 2022;25:213–25. 35115731 10.1038/s41593-021-00999-y PMC8825285

[vbag038-B14] Johnson ECB , Dammer EB, Duong DM et al Large-scale proteomic analysis of Alzheimer’s disease brain and cerebrospinal fluid reveals early changes in energy metabolism associated with microglia and astrocyte activation. Nat Med 2020;26:769–80. 32284590 10.1038/s41591-020-0815-6 PMC7405761

[vbag038-B15] Junker B , Schreiber F. Analysis of Biological Networks. NJ: John Wiley & Sons, 2008.

[vbag038-B16] Kuismin M , Sillanpää M. Estimation of covariance and precision matrix, network structure, and a view toward systems biology. WIREs Computational Stats 2017;9:1–13.

[vbag038-B17] Langfelder P , Horvath S. WGCNA: an R package for weighted correlation network analysis. BMC Bioinformatics 2008;9:559. 19114008 10.1186/1471-2105-9-559 PMC2631488

[vbag038-B18] Lauritzen S. Graphical Models. Oxford, UK: Oxford University Press, 1996.

[vbag038-B19] Lehtiö J , Arslan T, Siavelis I et al Proteogenomics of non-small cell lung cancer reveals molecular subtypes associated with specific therapeutic targets and immune evasion mechanisms. Nat Cancer 2021;2:1224–42. 34870237 10.1038/s43018-021-00259-9 PMC7612062

[vbag038-B20] Li C , Li H. Network-constrained regularization and variable selection for analysis of genomic data. Bioinformatics 2008;24:1175–82. 18310618 10.1093/bioinformatics/btn081

[vbag038-B21] Li T , Levina E, Zhu J. Prediction models for network-linked data. arXiv, 10.48550/arXiv.1602.01192, 2019, preprint: not peer reviewed.

[vbag038-B22] Liu Z , Meng X, Tang X et al Intratumoral tertiary lymphoid structures promote patient survival and immunotherapy response in head neck squamous cell carcinoma. Cancer Immunol Immunother 2023;72:1505–21. 36481914 10.1007/s00262-022-03310-5 PMC10198854

[vbag038-B23] Lung Cancer Cohort Consortium (LC3). The blood proteome of imminent lung cancer diagnosis. Nat Commun 2023;14:3042. 37264016 10.1038/s41467-023-37979-8 PMC10235023

[vbag038-B24] Manfredi M , Brandi J, Di Carlo C et al Mining cancer biology through bioinformatic analysis of proteomic data. Expert Rev Proteomics 2019;16:733–47. 31398064 10.1080/14789450.2019.1654862

[vbag038-B25] Åkesson J , Hojjati S, Hellberg S et al Proteomics reveal biomarkers for diagnosis, disease activity and long-term disability outcomes in multiple sclerosis. Nat Commun 2023;14:6903. 37903821 10.1038/s41467-023-42682-9 PMC10616092

[vbag038-B26] Matthews BW. Comparison of the predicted and observed secondary structure of t4 phage lysozyme. Biochim Biophys Acta 1975;405:442–51. 1180967 10.1016/0005-2795(75)90109-9

[vbag038-B27] Meinshausen N , Bühlmann P. High-dimensional graphs and variable selection with the lasso. Ann Statist 2006;34:1436–62.

[vbag038-B28] Monti GS , Filzmoser P. Sparse least trimmed squares regression with compositional covariates for high-dimensional data. Bioinformatics 2021;37:3805–14. 34358286 10.1093/bioinformatics/btab572

[vbag038-B29] Niu L , Thiele M, Geyer PE et al Noninvasive proteomic biomarkers for alcohol-related liver disease. Nat Med 2022;28:1277–87. 35654907 10.1038/s41591-022-01850-y PMC9205783

[vbag038-B30] Pandey A , Mann M. Proteomics to study genes and genomes. Nature 2000;405:837–46. 10866210 10.1038/35015709

[vbag038-B31] Peng J , Wang P, Zhou N et al Partial correlation estimation by joint sparse regression models. J Am Stat Assoc 2009;104:735–46. 19881892 10.1198/jasa.2009.0126 PMC2770199

[vbag038-B32] Petralia F , Ma W, Yaron T et al; Clinical Proteomic Tumor Analysis Consortium. Pan-cancer proteogenomics characterization of tumor immunity. Cell 2024;187:1255–77.e27. 38359819 10.1016/j.cell.2024.01.027 PMC10988632

[vbag038-B33] Qin Y , Liu H, Huang X et al GIMAP7 as a potential predictive marker for pan-cancer prognosis and immunotherapy efficacy. J Inflamm Res 2022;15:1047–61. 35210811 10.2147/JIR.S342503 PMC8858002

[vbag038-B34] Rappaport N , Twik M, Plaschkes I et al MalaCards: an amalgamated human disease compendium with diverse clinical and genetic annotation and structured search. Nucleic Acids Res 2017;45:D877–87. 27899610 10.1093/nar/gkw1012 PMC5210521

[vbag038-B35] Ren Y , Peterson CB, Vannucci M. Bayesian network-guided sparse regression with flexible varying effects. Biometrics 2024;80:ujae111. 39377518 10.1093/biomtc/ujae111

[vbag038-B36] Rozanova S , Barkovits K, Nikolov M et al Quantitative mass spectrometry-based proteomics: an overview. Methods Mol Biol 2021;2228:85–116. 33950486 10.1007/978-1-0716-1024-4_8

[vbag038-B37] Ruffin A , Cillo A, Tabib T et al B cell signatures and tertiary lymphoid structures contribute to outcome in head and neck squamous cell carcinomas. Nat Commun 2021;12:3349. 34099645 10.1038/s41467-021-23355-x PMC8184766

[vbag038-B38] Seal R , Braschi B, Gray K et al Genenames.org: the HGNC resources in 2023. Nucleic Acids Res 2023;51:D1003–9. 36243972 10.1093/nar/gkac888 PMC9825485

[vbag038-B39] Short MI , Fohner AE, Skjellegrind HK et al Proteome network analysis identifies potential biomarkers for brain aging. J Alzheimers Dis 2023;96:1767–80. 38007645 10.3233/JAD-230145 PMC10741337

[vbag038-B40] Shutta K , De Vito R, Scholtens D et al Gaussian graphical models with applications to omics analyses. Stat Med 2022;41:5150–87. 36161666 10.1002/sim.9546 PMC9672860

[vbag038-B41] Stelzer G , Rosen N, Plaschkes I et al The GeneCards suite: from gene data mining to disease genome sequence analyses. Curr Protoc Bioinformatics 2016;54:1.30.1–30.33. 10.1002/cpbi.5 27322403

[vbag038-B42] Tibshirani R. Regression shrinkage and selection via the lasso. J R Stat Soc Ser B 1996;58:267–88.

[vbag038-B43] Tutz G , Ulbricht J. Penalized regression with correlation-based penalty. Stat Comput 2009;19:239–53.

[vbag038-B44] Vella D , Zoppis I, Mauri G et al From protein-protein interactions to protein co-expression networks: a new perspective to evaluate large-scale proteomic data. EURASIP J Bioinform Syst Biol 2017;2017:6. 28477207 10.1186/s13637-017-0059-z PMC5359264

[vbag038-B45] Villanueva E , Smith T, Pizzinga M et al System-wide analysis of RNA and protein subcellular localization dynamics. Nat Methods 2024;21:60–71. 38036857 10.1038/s41592-023-02101-9 PMC10776395

[vbag038-B46] Wang Y , Li L, Li J et al Network modeling in biology: statistical methods for gene and brain networks. Stat Sci 2021;36:89–108. 34305304 10.1214/20-sts792 PMC8296984

[vbag038-B47] Weir C , Jan A. BMI Classification Percentile and Cut Off Points. Treasure Island, FL: StatPearls, 2023.

[vbag038-B48] Wisniewski J , Dus-Szachniewicz K, Ostasiewicz P et al Absolute proteome analysis of colorectal mucosa, adenoma, and cancer reveals drastic changes in fatty acid metabolism and plasma membrane transporters. J Proteome Res 2015;14:4005–18. 26245529 10.1021/acs.jproteome.5b00523

[vbag038-B49] Xu X , Khunsriraksakul C, Eales JM et al; Human Kidney Tissue Resource Study Group. Genetic imputation of kidney transcriptome, proteome and multi-omics illuminates new blood pressure and hypertension targets. Nat Commun 2024;15:2359. 38504097 10.1038/s41467-024-46132-y PMC10950894

[vbag038-B50] Yang W , Moon H, Kim H et al Proteomic approach reveals fkbp4 and s100a9 as potential prediction markers of therapeutic response to neoadjuvant chemotherapy in patients with breast cancer. J Proteome Res 2012;11:1078–88. 22074005 10.1021/pr2008187

[vbag038-B51] Yoshihara K , Shahmoradgoli M, Martinez E et al Inferring tumour purity and stromal and immune cell admixture from expression data. Nat Commun 2013;4:2612. 24113773 10.1038/ncomms3612 PMC3826632

[vbag038-B52] Yuan M , Lin Y. On the non-negative garrotte estimator. J R Stat Soc Ser B 2007;69:143–61.

[vbag038-B53] Zhang C , Zheng J, Lin Z et al Profiles of immune cell infiltration and immune-related genes in the tumor microenvironment of osteosarcoma. Aging (Albany NY) 2020;12:3486–501. 32039832 10.18632/aging.102824 PMC7066877

[vbag038-B54] Zou H. The adaptive lasso and its oracle properties. J Am Stat Assoc 2006;101:1418–29.

[vbag038-B55] Zou H , Hastie T. Regularization and variable selection via the elastic net. J R Stat Soc Ser B 2005;67:301–20.

[vbag038-B56] Zou H , Zhang H. On the adaptive elastic-net with a diverging number of parameters. Ann Stat 2009;37:1733–51. 20445770 10.1214/08-AOS625 PMC2864037

