# Supplementary material for: A network-guided penalized regression with application to proteomics data
Source: Bioinform Adv. 2026 Feb 3;6(1):vbag038. doi: 10.1093/bioadv/vbag038 (PMC12949433; doi:10.1093/bioadv/vbag038)
Supplement: vbag038_Supplementary_Data [file vbag038_supplementary_data.pdf]

Supplementary Material

Proof of Theorem 1. We first derive the joint asymptotic normality part. For any $\boldsymbol{\alpha} \in \mathbb{R}^t$, $\boldsymbol{\beta} \in \mathbb{R}^q$, denote $\boldsymbol{\theta} = (\boldsymbol{\alpha}^\mathsf{T}, \boldsymbol{\beta}^\mathsf{T})^\mathsf{T}$, $\hat{\boldsymbol{\theta}}_n = (\hat{\boldsymbol{\alpha}}_n^\mathsf{T}, \hat{\boldsymbol{\beta}}_n^\mathsf{T})^\mathsf{T}$, and $\boldsymbol{\theta}_0 = (\boldsymbol{\alpha}_0^\mathsf{T}, \boldsymbol{\beta}_0^\mathsf{T})^\mathsf{T}$. Define $\boldsymbol{u} = \sqrt{n}(\boldsymbol{\theta} - \boldsymbol{\theta}_0)$ and $\hat{\boldsymbol{u}}_n = \sqrt{n}(\hat{\boldsymbol{\theta}}_n - \boldsymbol{\theta}_0)$. Then the objective function $L_n(\boldsymbol{\alpha}, \boldsymbol{\beta})$ can be re-written as

$$L_n(\boldsymbol{u}) = \|Y - (\boldsymbol{U}, \boldsymbol{N})^\mathsf{T} \left(\boldsymbol{\theta}_0 + \frac{\boldsymbol{u}}{\sqrt{n}}\right) \|_2^2 + \lambda_n \sum_{s=t+1}^{t+q} \hat{w}_s \left|\theta_{0s} + \frac{u_s}{\sqrt{n}}\right|.$$

It is then easy to verify that $\hat{\boldsymbol{u}}_n = \arg\min_{\boldsymbol{u}} L_n(\boldsymbol{u})$. Note that $L_n(\boldsymbol{u}) - L_n(\boldsymbol{0}) = V_n(\boldsymbol{u})$ where

$$V_n(\boldsymbol{u}) = \boldsymbol{u}^\mathsf{T} n^{-1} \begin{pmatrix} \boldsymbol{U}^\mathsf{T}\boldsymbol{U} & \boldsymbol{U}^\mathsf{T}\boldsymbol{N} \\ \boldsymbol{N}^\mathsf{T}\boldsymbol{U} & \boldsymbol{N}^\mathsf{T}\boldsymbol{N} \end{pmatrix} \boldsymbol{u} - 2\frac{\epsilon^\mathsf{T}(\boldsymbol{U}, \boldsymbol{N})}{\sqrt{n}}\boldsymbol{u} + \frac{\lambda_n}{\sqrt{n}} \sum_{s=t+1}^{t+q} \hat{w}_s \sqrt{n} \left(\left|\theta_{0s} + \frac{u_s}{\sqrt{n}}\right| - |\theta_{0s}|\right).$$

The first term in the above display converges to $\boldsymbol{u}^\mathsf{T}\boldsymbol{C}\boldsymbol{u}$ for every $\boldsymbol{u}$. For the second term, by the Central Limit Theorem, we obtain $\epsilon^\mathsf{T}(\boldsymbol{U}, \boldsymbol{N})/\sqrt{n} \to_d \boldsymbol{W} = N(0, \sigma^2\boldsymbol{C})$. The limiting behavior of the last term depends on whether θ_{0s} is active or not for $s = t+1, \ldots, t+q$, which is equivalent to as whether β_{0j} is active or not for $j = 1, \ldots, q$. Note that if $\beta_{0j} \neq 0$, then $\tilde{\beta}_{nj} \to_p \beta_{0j}$ and $\hat{w}_j = |\tilde{\beta}_{nj}|^{-\nu} \to_p |\beta_{0j}|^{-\nu}$ by the Continuous Mapping Theorem. Also, $\sqrt{n}\left(\left|\beta_{0j} + \frac{u_j}{\sqrt{n}}\right| - |\beta_{0j}|\right) = u_j \text{sgn}(\beta_{0j})$. Hence, by Slutsky's Theorem, $\frac{\lambda_n}{\sqrt{n}}\hat{w}_j\sqrt{n}\left(\left|\beta_{0j} + \frac{u_j}{\sqrt{n}}\right| - |\beta_{0j}|\right) \to_p 0$. If $\beta_{0j} = 0$, then $\sqrt{n}\left(\left|\beta_{0j} + \frac{u_j}{\sqrt{n}}\right| - |\beta_{0j}|\right) = |u_j|$ and $\frac{\lambda_n}{\sqrt{n}}\hat{w}_j = \frac{\lambda_n}{\sqrt{n}} n^{\nu/2}(|\sqrt{n}\tilde{\beta}_{nj}|)^{-\nu} \to \infty$ since $\sqrt{n}\tilde{\beta}_{nj} = O_p(1)$. Therefore, the last term converges in probability to 0 if $\theta_{0s} \neq 0$, and it converges to ∞ if $\theta_{0s} = 0$. Now let $\mathcal{S} = \{1, 2, \ldots, t\} \cup \{s : \theta_{0s} \neq 0,\, s = t+1, \ldots, t+q\}$. Then by Slutsky's Theorem, we get $V_n(\boldsymbol{u}) \to_d V(\boldsymbol{u})$ for every $\boldsymbol{u}$, where

$$V(\boldsymbol{u}) = \begin{cases} \boldsymbol{u}_\mathcal{S}^\mathsf{T}\boldsymbol{C}_\mathcal{S}\boldsymbol{u}_\mathcal{S} - 2\boldsymbol{u}_\mathcal{S}^\mathsf{T}\boldsymbol{W}_\mathcal{S} & \text{if } u_s = 0 \text{ for } s \notin \mathcal{S} \\ \infty & \text{otherwise} \end{cases}$$

Note that $V(\boldsymbol{u})$ is convex and the minimum of $V(\boldsymbol{u})$ is uniquely achieved at $(\boldsymbol{C}_\mathcal{S}^{-1}\boldsymbol{W}_\mathcal{S}, \boldsymbol{0})^\mathsf{T}$ where $\boldsymbol{C}_\mathcal{S}^{-1}\boldsymbol{W}_\mathcal{S} \in \mathbb{R}^{t+r}$ and $\boldsymbol{0} \in \mathbb{R}^{q-r}$. By the epi-convergence results of Geyer (1994) and Knight and Fu

(2000), we have

$$\hat{\boldsymbol{u}}_{n\mathcal{S}} \to_d \boldsymbol{C}_{\mathcal{S}}^{-1}\boldsymbol{W}_{\mathcal{S}} \quad \text{and} \quad \hat{\boldsymbol{u}}_{n\mathcal{S}^c} \to_d \boldsymbol{0}. \tag{1}$$

Therefore, $\hat{\boldsymbol{u}}_{n\mathcal{S}} = \sqrt{n}\binom{\hat{\boldsymbol{\alpha}}_n - \boldsymbol{\alpha}_0}{\hat{\boldsymbol{\beta}}_{n\mathcal{J}} - \boldsymbol{\beta}_{0\mathcal{J}}} \to_d \boldsymbol{C}_{\mathcal{S}}^{-1}\boldsymbol{W}_{\mathcal{S}} = N(\boldsymbol{0}, \sigma^2\boldsymbol{C}_{\mathcal{S}}^{-1})$, where $\boldsymbol{C}_{\mathcal{S}} \in \mathbb{R}^{(t+r)\times(t+r)}$ is the top-left block matrix (i.e., sub-matrix) of $\boldsymbol{C} \in \mathbb{R}^{(t+q)\times(t+q)}$.

Now we show the consistency part. Note that the asymptotic normality results imply that $\hat{\boldsymbol{\alpha}}_n \to_p \boldsymbol{\alpha}_0$ and $\hat{\beta}_{nj} \to_p \beta_{0j}$ for $\forall j \in \mathcal{J}$, and hence $P(j \in \hat{\mathcal{J}}_n) \to 1$. Then it suffices to show that $\forall j' \notin \mathcal{J}$, $P(j' \in \hat{\mathcal{J}}_n) \to 0$. When $j' \in \hat{\mathcal{J}}_n$, we observe that $2N_{j'}^{\mathsf{T}}(Y - \boldsymbol{U}\hat{\boldsymbol{\alpha}}_n - \boldsymbol{N}\hat{\boldsymbol{\beta}}_n) = \lambda_n \hat{w}_{j'}\text{sgn}(\hat{\beta}_{nj'})$ by the Karush–Kuhn–Tucker (KKT) conditions. Note that $\lambda_n \hat{w}_{j'}\text{sgn}(\hat{\beta}_{nj'})/\sqrt{n} = \frac{\lambda_n}{\sqrt{n}} n^{\nu/2} \frac{1}{|\sqrt{n}\hat{\beta}_{nj'}|^{\nu}}\text{sgn}(\hat{\beta}_{nj'}) \to_p \infty$, whereas

$$\begin{aligned} 2N_{j'}^{\mathsf{T}}(Y - \boldsymbol{U}\hat{\boldsymbol{\alpha}}_n - \boldsymbol{N}\hat{\boldsymbol{\beta}}_n)/\sqrt{n} &= 2N_{j'}^{\mathsf{T}}\boldsymbol{U}\sqrt{n}(\boldsymbol{\alpha}_0 - \hat{\boldsymbol{\alpha}}_n)/n \\ &\quad + 2N_{j'}^{\mathsf{T}}\boldsymbol{N}\sqrt{n}(\boldsymbol{\beta}_0 - \hat{\boldsymbol{\beta}}_n)/n + 2N_{j'}^{\mathsf{T}}\epsilon/\sqrt{n}. \end{aligned}$$

By (1) and Slutsky's Theorem, we know that $2N_{j'}^{\mathsf{T}}\boldsymbol{U}\sqrt{n}(\boldsymbol{\alpha}_0 - \hat{\boldsymbol{\alpha}}_n)/n$ and $2N_{j'}^{\mathsf{T}}\boldsymbol{N}\sqrt{n}(\boldsymbol{\beta}_0 - \hat{\boldsymbol{\beta}}_n)/n$ converges in distribution to some normal distribution and $2N_{j'}^{\mathsf{T}}\epsilon/\sqrt{n} \to_d N(\boldsymbol{0}, 4\|N_{j'}\|_2^2\sigma^2))$. Hence, $P(j' \in \hat{\mathcal{J}}_n) \le P\left(2N_{j'}^{\mathsf{T}}(Y - \boldsymbol{U}\hat{\boldsymbol{\alpha}}_n - \boldsymbol{N}\hat{\boldsymbol{\beta}}_n) = \lambda_n \hat{w}_{j'}\text{sgn}(\hat{\beta}_{nj'})\right) \to 0$. This proves the consistency part.

References

Geyer, C. (1994). On the asymptotics of constrained m-estimation. *Annals of Statistics*, *22*, 1993–2010.

Knight, K., & Fu, W. (2000). Asymptotics for lasso-type estimators. *Annals of Statistics*, *28*(5), 1356–1378.

Supplementary Table 1: Performance results of methods applied to CPTAC-HNSCC patients using 100 repeated train/test splits. The averages are provided with the standard deviations in parentheses. The best results are highlighted in boldface.

	RMSE	CSL
NG ($\delta = 0.01$)	**1.96** (0.29)	**0.91** (0.17)
NG ($\delta = 0.02$)	**1.96** (0.29)	0.89 (0.16)
NG ($\delta = 0.03$)	**1.96** (0.30)	0.85 (0.16)
aLasso	2.10 (0.30)	1.15 (0.40)
Lasso	2.18 (0.31)	1.15 (0.40)
Ridge	2.24 (0.27)	2.11 (0.88)
enet	2.18 (0.33)	1.95 (1.03)
CBPE	2.79 (0.33)	0.49 (0.14)
SLS	2.97 (3.25)	2.27 (0.39)

δ is the proportion used for the number of hub protein nodes in a network

Supplementary Table 2: Simulation results under strong signal case using betweenness centrality for network-guided (NG) method. The best results are highlighted in boldface.

Setting	n	p	Method	RMSE	CSL	F1 score	MCC	Avg. runtime (sec)
I	50	60	NG ($\delta = 0.06$)	1.35 (0.50)	**1.01** (0.01)	0.81 (0.11)	0.76 (0.14)	0.53
			NG ($\delta = 0.08$)	1.31 (0.46)	**1.01** (0.01)	0.83 (0.10)	0.79 (0.13)	0.53
			NG ($\delta = 0.10$)	**1.33** (0.49)	**1.01** (0.01)	0.81 (0.10)	0.76 (0.12)	0.53
			aLasso	2.89 (0.78)	1.04 (0.03)	0.71 (0.15)	0.66 (0.19)	0.04
			Lasso	1.88 (0.71)	1.03 (0.02)	0.64 (0.10)	0.56 (0.14)	0.02
			enet	2.22 (0.58)	1.04 (0.02)	0.52 (0.07)	0.40 (0.11)	0.02
			ridge	8.16 (0.72)	1.63 (0.14)	0.34 (0.00)	–	0.02
			CBPE	2.77 (0.31)	1.04 (0.03)	0.34 (0.00)	–	0.36
			SLS	7.15 (2.06)	0.75 (0.07)	**0.89** (0.11)	**0.87** (0.14)	0.24
II	100	60	NG ($\delta = 0.06$)	0.67 (0.08)	1.01 (0.00)	**0.99** (0.04)	**0.98** (0.05)	0.52
			NG ($\delta = 0.08$)	**0.66** (0.09)	**1.00** (0.00)	**0.99** (0.04)	**0.98** (0.05)	0.51
			NG ($\delta = 0.10$)	0.67 (0.09)	**1.00** (0.00)	0.96 (0.02)	0.95 (0.03)	0.51
			aLasso	0.70 (0.10)	1.01 (0.00)	0.98 (0.04)	**0.98** (0.04)	0.02
			Lasso	0.74 (0.12)	1.02 (0.00)	0.71 (0.07)	0.65 (0.08)	0.01
			enet	0.89 (0.14)	1.02 (0.00)	0.51 (0.05)	0.41 (0.07)	0.01
			ridge	0.94 (0.11)	1.02 (0.01)	0.34 (0.00)	–	0.02
			CBPE	1.57 (0.17)	1.02 (0.01)	0.34 (0.00)	–	0.19
			SLS	7.15 (1.73)	0.73 (0.05)	0.94 (0.05)	0.93 (0.06)	0.28
III	100	300	NG ($\delta = 0.01$)	**1.10** (0.13)	**1.00** (0.00)	**0.97** (0.03)	**0.97** (0.03)	0.95
			NG ($\delta = 0.02$)	1.15 (0.13)	**1.00** (0.00)	0.88 (0.03)	0.88 (0.03)	0.95
			NG ($\delta = 0.03$)	1.18 (0.13)	**1.00** (0.00)	0.80 (0.03)	0.79 (0.03)	0.95
			aLasso	2.42 (0.46)	1.02 (0.01)	0.85 (0.06)	0.86 (0.05)	0.03
			Lasso	1.15 (0.21)	1.02 (0.00)	0.92 (0.06)	0.92 (0.05)	0.02
			enet	1.22 (0.23)	1.02 (0.00)	0.85 (0.07)	0.85 (0.07)	0.03
			ridge	9.87 (0.90)	1.45 (0.07)	0.08 (0.00)	–	0.13
			CBPE	5.29 (0.33)	1.07 (0.02)	0.08 (0.00)	–	8.83
			SLS	26.5 (7.73)	0.54 (0.13)	0.86 (0.28)	0.85 (0.34)	1.32

δ is the proportion used for the number of hub protein nodes in a network

Supplementary Table 3: Simulation results under weak signal case using betweenness centrality for network-guided (NG) method. The best results are highlighted in boldface.

Setting	n	p	Method	RMSE	CSL	F1 score	MCC	Avg. runtime (sec)
I	50	60	NG ($\delta = 0.06$)	0.23 (0.09)	**1.01** (0.02)	**0.90** (0.06)	**0.89** (0.07)	0.52
			NG ($\delta = 0.08$)	0.19 (0.07)	**1.01** (0.01)	**0.90** (0.04)	0.88 (0.04)	0.52
			NG ($\delta = 0.10$)	**0.18** (0.05)	**1.01** (0.01)	0.86 (0.04)	0.84 (0.04)	0.53
			aLasso	0.49 (0.11)	1.07 (0.04)	0.80 (0.05)	0.79 (0.06)	0.03
			Lasso	0.34 (0.14)	1.05 (0.03)	0.62 (0.09)	0.58 (0.11)	0.02
			enet	0.46 (0.14)	1.07 (0.04)	0.53 (0.07)	0.48 (0.09)	0.02
			ridge	2.06 (0.11)	3.43 (3.41)	0.25 (0.00)	–	0.02
			CBPE	0.89 (0.12)	0.97 (0.04)	0.25 (0.00)	–	0.34
			SLS	1.89 (0.27)	1.27 (0.16)	0.67 (0.13)	0.62 (0.14)	0.20
II	100	60	NG ($\delta = 0.06$)	**0.08** (0.01)	**1.01** (0.00)	0.96 (0.02)	0.96 (0.03)	0.52
			NG ($\delta = 0.08$)	**0.08** (0.01)	**1.01** (0.00)	0.95 (0.01)	0.94 (0.01)	0.52
			NG ($\delta = 0.10$)	**0.08** (0.01)	**1.01** (0.00)	0.90 (0.02)	0.89 (0.02)	0.52
			aLasso	**0.08** (0.01)	**1.01** (0.00)	**1.00** (0.00)	**1.00** (0.00)	0.02
			Lasso	**0.08** (0.01)	1.02 (0.00)	0.78 (0.07)	0.77 (0.07)	0.01
			enet	0.09 (0.01)	1.02 (0.00)	0.64 (0.07)	0.62 (0.07)	0.01
			ridge	0.54 (0.05)	1.10 (0.03)	0.25 (0.00)	–	0.02
			CBPE	0.36 (0.04)	0.96 (0.01)	0.25 (0.00)	–	0.21
			SLS	1.82 (0.17)	1.23 (0.09)	0.75 (0.08)	0.71 (0.09)	0.26
III	100	300	NG ($\delta = 0.01$)	0.27 (0.09)	**1.01** (0.01)	**0.88** (0.06)	**0.88** (0.07)	0.98
			NG ($\delta = 0.02$)	**0.19** (0.06)	**1.01** (0.01)	0.81 (0.02)	0.80 (0.02)	0.98
			NG ($\delta = 0.03$)	**0.19** (0.07)	**1.01** (0.01)	0.70 (0.02)	0.71 (0.03)	0.98
			aLasso	0.45 (0.06)	1.05 (0.02)	0.81 (0.03)	0.82 (0.03)	0.05
			Lasso	0.46 (0.05)	1.06 (0.02)	0.44 (0.09)	0.47 (0.07)	0.03
			enet	0.53 (0.07)	1.08 (0.03)	0.33 (0.06)	0.39 (0.05)	0.03
			ridge	2.14 (0.12)	1.83 (0.60)	0.06 (0.00)	–	0.14
			CBPE	2.48 (0.22)	0.73 (0.07)	0.06 (0.00)	–	8.78
			SLS	2.37 (0.25)	0.66 (0.07)	0.67 (0.12)	0.68 (0.10)	1.12

δ is the proportion used for the number of hub protein nodes in a network

Supplementary Table 4: Simulation results under strong signal case using eigenvector centrality for network-guided (NG) method. The best results are highlighted in boldface.

Setting	n	p	Method	RMSE	CSL	F1 score	MCC	Avg. runtime (sec)
I	50	60	NG ($\delta = 0.06$)	1.34 (0.50)	**1.01** (0.01)	0.81 (0.11)	0.76 (0.14)	0.54
			NG ($\delta = 0.08$)	1.29 (0.44)	**1.01** (0.01)	0.83 (0.10)	0.79 (0.13)	0.54
			NG ($\delta = 0.10$)	**1.27** (0.45)	**1.01** (0.01)	0.82 (0.09)	0.77 (0.11)	0.54
			aLasso	2.89 (0.78)	1.04 (0.03)	0.71 (0.15)	0.66 (0.19)	0.04
			Lasso	1.88 (0.71)	1.03 (0.02)	0.64 (0.10)	0.56 (0.14)	0.02
			enet	2.22 (0.58)	1.04 (0.02)	0.52 (0.07)	0.40 (0.11)	0.02
			ridge	8.16 (0.72)	1.63 (0.14)	0.34 (0.00)	–	0.02
			CBPE	2.77 (0.31)	1.04 (0.03)	0.34 (0.00)	–	0.36
			SLS	7.15 (2.06)	0.75 (0.07)	**0.89** (0.11)	**0.87** (0.14)	0.24
II	100	60	NG ($\delta = 0.06$)	**0.66** (0.09)	1.01 (0.00)	**0.99** (0.04)	0.98 (0.04)	0.51
			NG ($\delta = 0.08$)	**0.66** (0.09)	1.01 (0.00)	**0.99** (0.04)	**0.99** (0.04)	0.50
			NG ($\delta = 0.10$)	0.67 (0.10)	**1.00** (0.00)	0.96 (0.03)	0.95 (0.03)	0.51
			aLasso	0.70 (0.10)	1.01 (0.00)	0.98 (0.04)	0.98 (0.04)	0.02
			Lasso	0.74 (0.12)	1.02 (0.00)	0.71 (0.07)	0.65 (0.08)	0.01
			enet	0.89 (0.14)	1.02 (0.00)	0.51 (0.05)	0.41 (0.07)	0.01
			ridge	0.94 (0.11)	1.02 (0.01)	0.34 (0.00)	–	0.02
			CBPE	1.57 (0.17)	1.02 (0.01)	0.34 (0.00)	–	0.19
			SLS	7.15 (1.73)	0.73 (0.05)	0.94 (0.05)	0.93 (0.06)	0.28
III	100	300	NG ($\delta = 0.01$)	**1.11** (0.13)	**1.00** (0.00)	**0.97** (0.03)	**0.97** (0.03)	0.98
			NG ($\delta = 0.02$)	1.14 (0.13)	**1.00** (0.00)	0.88 (0.03)	0.87 (0.03)	0.98
			NG ($\delta = 0.03$)	1.18 (0.14)	**1.00** (0.00)	0.79 (0.03)	0.79 (0.03)	0.99
			aLasso	2.42 (0.46)	1.02 (0.01)	0.85 (0.06)	0.86 (0.05)	0.03
			Lasso	1.15 (0.21)	1.02 (0.00)	0.92 (0.06)	0.92 (0.05)	0.02
			enet	1.22 (0.23)	1.02 (0.00)	0.85 (0.07)	0.85 (0.07)	0.03
			ridge	9.87 (0.90)	1.45 (0.07)	0.08 (0.00)	–	0.13
			CBPE	5.29 (0.33)	1.07 (0.02)	0.08 (0.00)	–	8.83
			SLS	26.5 (7.73)	0.54 (0.13)	0.86 (0.28)	0.85 (0.34)	1.32

δ is the proportion used for the number of hub protein nodes in a network

Supplementary Table 5: Simulation results under weak signal case using eigenvector centrality for network-guided (NG) method. The best results are highlighted in boldface.

Setting	n	p	Method	RMSE	CSL	F1 score	MCC	Avg. runtime (sec)
I	50	60	NG ($\delta = 0.06$)	0.24 (0.10)	1.02 (0.02)	**0.90** (0.06)	0.88 (0.07)	0.52
			NG ($\delta = 0.08$)	**0.18** (0.04)	**1.01** (0.01)	**0.90** (0.03)	**0.89** (0.04)	0.52
			NG ($\delta = 0.10$)	**0.18** (0.04)	**1.01** (0.01)	0.86 (0.03)	0.83 (0.04)	0.53
			aLasso	0.49 (0.11)	1.07 (0.04)	0.80 (0.05)	0.79 (0.06)	0.03
			Lasso	0.34 (0.14)	1.05 (0.03)	0.62 (0.09)	0.58 (0.11)	0.02
			enet	0.46 (0.14)	1.07 (0.04)	0.53 (0.07)	0.48 (0.09)	0.02
			ridge	2.06 (0.11)	3.43 (3.41)	0.25 (0.00)	–	0.02
			CBPE	0.89 (0.12)	0.97 (0.04)	0.25 (0.00)	–	0.34
			SLS	1.89 (0.27)	1.27 (0.16)	0.67 (0.13)	0.62 (0.14)	0.20
II	100	60	NG ($\delta = 0.06$)	**0.08** (0.01)	**1.01** (0.00)	0.96 (0.02)	0.96 (0.03)	0.51
			NG ($\delta = 0.08$)	**0.08** (0.01)	**1.01** (0.00)	0.95 (0.00)	0.94 (0.00)	0.51
			NG ($\delta = 0.10$)	**0.08** (0.01)	**1.01** (0.00)	0.90 (0.01)	0.89 (0.01)	0.51
			aLasso	**0.08** (0.01)	**1.01** (0.00)	**1.00** (0.00)	**1.00** (0.00)	0.02
			Lasso	**0.08** (0.01)	1.02 (0.00)	0.78 (0.07)	0.77 (0.07)	0.01
			enet	0.09 (0.01)	1.02 (0.00)	0.64 (0.07)	0.62 (0.07)	0.01
			ridge	0.54 (0.05)	1.10 (0.03)	0.25 (0.00)	–	0.02
			CBPE	0.36 (0.04)	0.96 (0.01)	0.25 (0.00)	–	0.21
			SLS	1.82 (0.17)	1.23 (0.09)	0.75 (0.08)	0.71 (0.09)	0.26
III	100	300	NG ($\delta = 0.01$)	0.28 (0.09)	**1.01** (0.01)	**0.88** (0.06)	**0.88** (0.06)	0.95
			NG ($\delta = 0.02$)	**0.19** (0.06)	**1.01** (0.01)	0.81 (0.03)	0.81 (0.03)	0.96
			NG ($\delta = 0.03$)	**0.19** (0.06)	**1.01** (0.01)	0.70 (0.03)	0.71 (0.03)	0.96
			aLasso	0.45 (0.06)	1.05 (0.02)	0.81 (0.03)	0.82 (0.03)	0.05
			Lasso	0.46 (0.05)	1.06 (0.02)	0.44 (0.09)	0.47 (0.07)	0.03
			enet	0.53 (0.07)	1.08 (0.03)	0.33 (0.06)	0.39 (0.05)	0.03
			ridge	2.14 (0.12)	1.83 (0.60)	0.06 (0.00)	–	0.14
			CBPE	2.48 (0.22)	0.73 (0.07)	0.06 (0.00)	–	8.78
			SLS	2.37 (0.25)	0.66 (0.07)	0.67 (0.12)	0.68 (0.10)	1.12

δ is the proportion used for the number of hub protein nodes in a network

Supplementary Table 6: Regression coefficients of hub proteins under different values of δ from the final fitted model using our proposed approach, adjusted for covariates and selected non-hubs. Gene symbols are approved by the HUGO Gene Nomenclature Committee (HGNC). Proteins selected under all three values of δ are boldfaced.

Gene Symbol	NG ($\delta = 0.01$)	NG ($\delta = 0.02$)	NG ($\delta = 0.03$)
**PABPC1**	-0.381	-0.276	-0.141
**LGALS1**	0.766	0.808	0.784
**GIMAP7**	0.655	0.516	-0.018
MEN1		-0.520	-0.352
RPLP1		-0.280	-0.387
HNRNPD		0.247	0.408
CASP10			0.461
BLNK			0.594
SDC1			-0.198
MUC4			-0.314

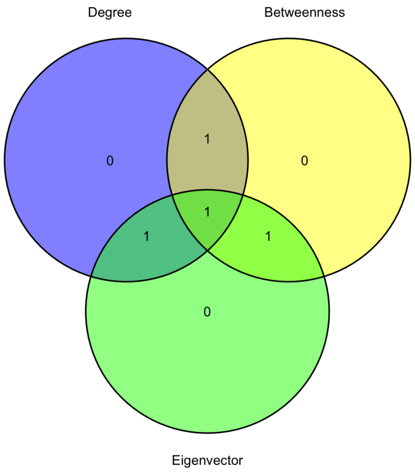

(a)

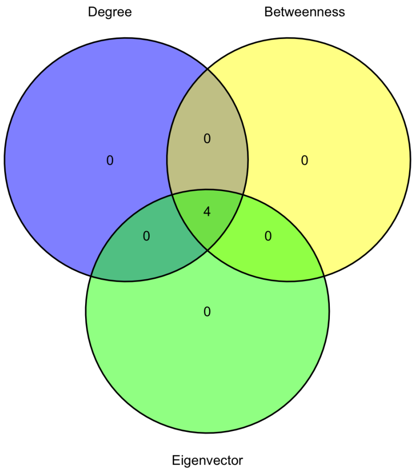

(b)

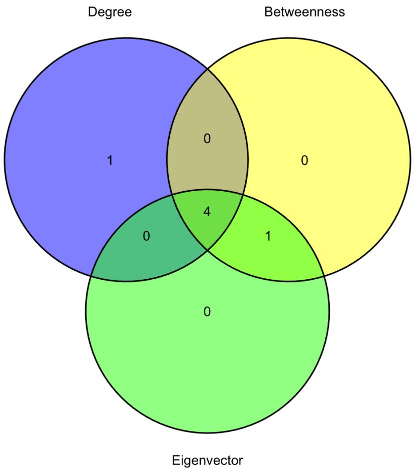

(c)

Supplementary Figure 1: An example of a Venn diagram from a single Monte Carlo simulation replicate with $n = 50$ and $p = 60$, showing hubs identified using different network properties for (a) $\delta = 0.06$, (b) $\delta = 0.08$, and (c) $\delta = 0.10$.
